# First Report of Cattle Tick *Rhipicephalus* (*Boophilus*) *annulatus* in Egypt Resistant to Ivermectin

**DOI:** 10.3390/insects10110404

**Published:** 2019-11-15

**Authors:** Saeed El-Ashram, Shawky M. Aboelhadid, Asmaa A. Kamel, Lilian N. Mahrous, Magdy M. Fahmy

**Affiliations:** 1 School of Life Science and Engineering, Foshan University, Foshan 528231, China; 2 Faculty of Science, Kafrelsheikh University, Kafr el-Sheikh 33516, Egypt; 3 Department of Parasitology, Faculty of Veterinary Medicine, Beni-Suef University, Beni-Suef 62515, Egypt; drasmaaalaa@yahoo.com (A.A.K.); Lilian_nagy@yahoo.com (L.N.M.); 4 Department of Parasitology, Faculty of Veterinary Medicine, Cairo University, Cairo 11865, Egypt; mmfahmy@hotmail.com

**Keywords:** *Rhipicephalus annulatus*, larval immersion test, ivermectin, resistance

## Abstract

Tick control is mainly dependent on the application of acaricides, but resistance has developed to almost all classes of acaricides, including macrolactones. Therefore, we aimed to investigate ivermectin resistance among tick populations in middle Egypt. The larval immersion test was conducted using a commercial formulation of ivermectin (1%). Different concentrations of the immersion solution (0.0000625% (625 × 10^−7^%), 0.000125% (125 × 10^−6^%), 0.0005% (5 × 10^−4^%), 0.001% (1 × 10^−3^%), 0.0025% (2.5 × 10^−3^%), 0.005% (5 × 10^−3^), and 0.01% (1 × 10^−2^%)) were prepared by diluting a commercial ivermectin (1%) with distilled water containing 1% (*v*/*v*) ethanol and 2% (*v*/*v*) TritonX-100. Field populations of *Rhipicephalus annulatus* were collected from five different localities in Beni-Suef province, Egypt. Adult engorged female ticks were collected and assessed for oviposition and egg fertility. Eggs were collected, and hatched larvae were then used in the experiment. Application of acaricides was conducted on 10-day-old larvae. There was a significant difference in the LC_50_ (50% lethal concentration) among the examined localities on the log dose-response plot, where, the LC_50_ of tick populations from two localities (Emin elaros and Aldiabia) was higher than the other localities (Alhalabia, Alkom, and Beshnna). Besides, tick populations from Emin elaros and Aldiabia showed higher LC_90_ values with lower slope values compared to those from Alhalabia, Alkom, and Beshnna. According to these values (LC_50_, LC_90_, and slope values), as well as a history of acaricide failure to ticks in these areas, *R. annulatus* developed resistance to ivermectin. This study documents the first report of field populations of *R. annulatus* resistant to ivermectin in Egypt.

## 1. Introduction

Hard ticks are the most common cattle ectoparasites that are distributed worldwide. The chemical control of such parasites is mainly through acaricides, which are the primary method used for tick control. However, the widespread use of chemical acaricides has resulted in the selection of acaricide-resistant ticks. Acaricide resistance can be defined as a significant increase in the number of individuals within a tick population that can tolerate doses of acaricides proved to be lethal for the majority of individuals in a sensitive population of the same species [1]. Acaricide resistance continues to be a major obstacle in the control of cattle ticks in many countries, such as Mexico, Australia, and Brazil [2,3]. Ivermectin is one of the macrocyclic lactones (MLs), which are widely used acaricides for controlling tick populations and nematodes. Ticks and nematodes represent the most important constraints to livestock production, particularly in tropical and subtropical areas [4,5]. However, the improper use of macrocyclic lactones has been associated with the development of resistance in populations of ticks worldwide [6,7]. Pharmacokinetics of such compounds (i.e., MLs) in arthropods is well understood by their high affinity to glutamate-gated chloride channels (Glu-Cl) that are found in muscle and nerves. The inhibition of these channels causes a slow and irreversible membrane conductance, resulting in a somatic musculature paralysis and a consequent death of the parasite [8]. Besides, adenosine triphosphate binding cassette (ABC) transporters and esterases play a major role in the detoxification of ivermectin [9]. The early detection of resistance is the target to avoid further selection of resistant ticks using the same active ingredient and to delay the subsequent spread of ivermectin resistance. The larval immersion test (LIT) has been recommended by Food and Agriculture Organization (FAO) as a standard bioassay for testing resistance to MLs [10], which was modified by Klafke et al. [6] and Sabatini et al. [11] to determine 50% and 99.9% lethal concentrations. Several reports have recorded ivermectin resistance in *Rhipicephalus microplus* populations in Mexico by Perez-Cogollo [12], Uruguay by Castro-Janer [13], and in Brazil by Klafke et al. [6]. In a previous study, we showed that tick populations were resistant to deltamethrin in the same study areas (Beni-Suef, Egypt) [14]. Therefore, this study aimed to evaluate the resistance of field populations of *Boophilus annulatus* in Beni-Suef province, Egypt, to ivermectin.

## 2. Materials and Methods

This study was conducted according to the ethical standards of the Faculty of Veterinary Medicine, Beni-Suef University, Egypt, and approved by the Institutional Animal Care and Use Committee of Beni-Suef University (2018-BSUV22).

### 2.1. Study Area and Data Collection from Farmers

**Figure 1:**
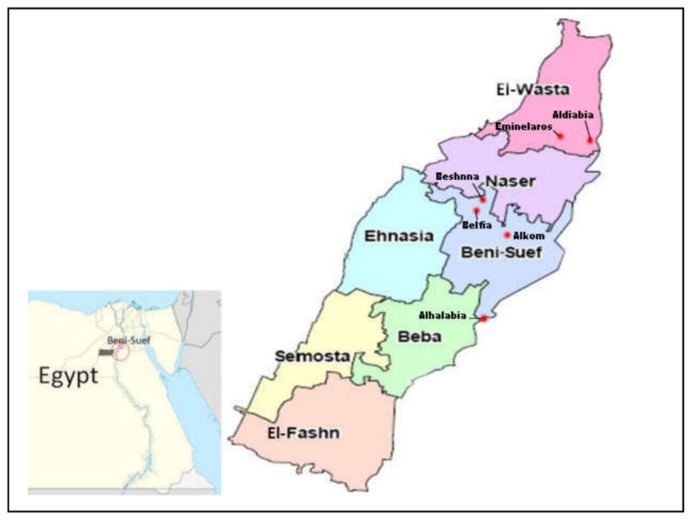
Map of the study area within Beni-Suef province, Egypt. Red dots indicate tick collection sites.

This study was carried out at five different localities, including Alhalabia, Alkom, Beshnna, Aldiabia, and Emin elaros in Beni-Suef province, Egypt (GPS coordinates: 29°04′ N 31°05′ E) (Figure 1). Farmers in these localities were interviewed and questioned to obtain data concerning whether their ivermectin use was mainly as an acaricide or anthelminthic. Furthermore, we queried the farmers as to whether ticks or parasitic worms were more of a problem in their livestock.

### 2.2. Collection of Ticks and Identification

Fifty engorged females were collected [15] directly from naturally infested cattle in each locality, placed in identified cotton plugged bottles, and transported to the Laboratory of Parasitology, Faculty of Veterinary Medicine, Beni-Suef University for identification under a stereomicroscope using the keys given by Hoogstraal and Kaiser [16] and Walker et al. [17].

### 2.3. Preparation of R. annulatus larvae

The collected female ticks (*R.* (*B.*) *annulatus*) from a particular area were designated as an isolate. Ticks were thoroughly washed in distilled water, allowed to dry on paper towels, labeled, and incubated individually in separate cotton plugged tubes (2.5 cm diameter) at 27–28 °C with 80–90% relative humidity and a 12-h light/dark cycle for oviposition. After 14 days of egg-laying, the females were discarded, and the number of eggs in each tube was counted. Eggs were hatched within 2 weeks upon removal from the tube. The 10-day-old hatched larvae were ready for testing.

### 2.4. Preparation of Ivermectin for In Vitro Application

A commercial formulation of ivermectin 1% (Normectin^®^ 1%) was diluted with distilled water containing 1% (*v*/*v*) ethanol and 2% (*v*/*v*) TritonX-100 solution (Eth-TX) to prepare immersion solutions. Different concentrations of the prepared ivermectin were used (0.0000625% (625 × 10^−7^%), 0.000125% (125 × 10^−6^%), 0.0005% (5 × 10^−4^%), 0.001% (1 × 10^−3^%), 0.0025% (2.5 × 10^−3^%), 0.005% (5 × 10^−3^), and 0.01% (1 × 10^−2^%)).

### 2.5. Larval Immersion Test (LIT)

This assay was done in a 1.5 mL microcentrifuge tube, in which 1 mL of each concentration was added. Larvae (200 mg of larvae = 400 larvae) were subsequently added by a paintbrush. A solution without ivermectin was prepared by adding 10 µL of Eth-TX solution to 990 µL of distilled water and served as the control. The tubes, which were closed and vigorously shaken for 15 s, were then gently shaken for 10 min [2]. After treatment, the larvae were transferred to filter paper, dried, then again transferred to a filter paper (8.5 × 7.5 cm), which was folded forming a packet, and closed with ‘‘bulldog’’ clips. These packets were incubated in a biological oxygen demand (BOD) chamber (27–28 °C with 80–90% relative humidity). The mortality rate was determined by counting live and dead larvae (no locomotion). There were triplicates for each concentration.

### 2.6. Statistical Analysis

Data of the mortality rates were submitted to a Probit analysis. Chi-square test was used to test the hypothesis parallelism and equality (*p* = 0.05) with Polo-Plus software [18]. Lethal concentrations (LC) for 50% and 90%, with their respective confidence intervals of 95% (CI 95%), were calculated.

## 3. Results

### 3.1. History of Ivermectin Use at the Five Study Areas

The survey revealed that 90% of farmers in Aldiabia and Emin elaros areas relied mainly on ivermectin for the treatment of tick infestations. However, farmers in Alhalabia, Alkom, and Beshnna areas used an additional repellent spray in combination with ivermectin injection in heavily infested animals. Furthermore, farmers complained about repeated tick infestation of animals in the study area after a short period from the application of ivermectin (within ~15 days).

### 3.2. Larval Immersion Test Results

**Figure 2:**
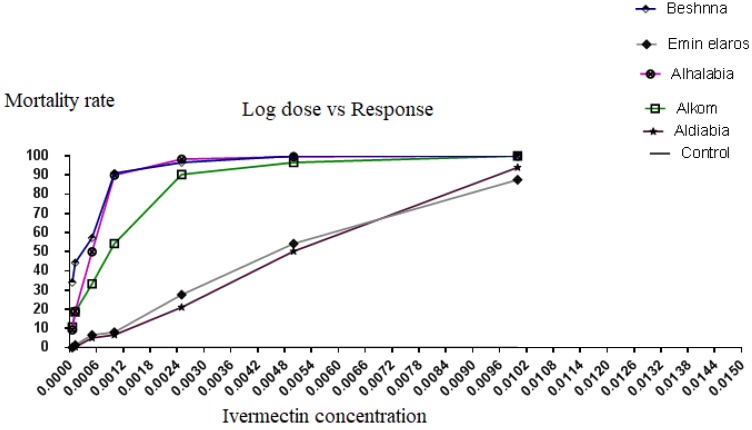
Ivermectin probit mortality × log concentration plots and regression lines of tick populations from the five study areas (Alhalabia, Alkom, Beshnna, Aldiabia, and Eminelaros).

The LC_50_ and LC_90_ values for ivermectin with their respective 95% confidence intervals (CI) and slopes for each population are shown in Table 1. The tick population from Aldiabia and Emin elaros areas, which relied on ivermectin treatment, had significantly higher LC_50_ and LC_90_ values than the other tick populations. The calculated LC_50_ (CI 95%) in Emin elaros and Aldiabia areas was 0.0052% (0.0037 to 0.0066) and 0.0051% (0.0046 to 0.0056), respectively. Furthermore, the value was 0.00052% (0.00017 to 0.00088), 0.00029% (0.000062 to 0.000498), and 0.0011% (0.00063 to 0.0015) in Alhalabia, Beshnna, and Alkom areas, respectively (Table 1). The tick populations from Emen elaros and Aldiabia areas could tolerate the higher doses of ivermectin, while those in Alhalabia and Beshnna areas were more susceptible and found dead at lower concentrations of ivermectin. LC_90_ (CI 95%) was 0.0084%, 0.0011%, 0.0013%, and 0.0027% for tick populations in Aldiabia, Alhalabia, Beshnna, and Alkom areas, respectively (Table 2). The hypothesis of parallelism and equality between regression lines for tick populations from Emen elaros and Aldiabia areas was neglected when compared to tick populations from the other three study areas (Figure 2).

## 4. Discussion

A few decades ago, the first macrolactones were introduced into the market and continue to be one of the major alternatives for control of bovine parasites, especially in cases of parasites resistant to other chemical groups [19]. *R. microplus*, which is resistant to macrolactones, has been documented worldwide [6,20]. The dose-response bioassays and/or assessment of the field efficacy of acaricides are the approved methods for confirmation of resistance (FAO, 2004, the World Association for the Advancement of Veterinary Parasitology (WAAVP) [20].

In the present study, various levels of ivermectin susceptibility/resistance in five populations of ticks were reported in Beni-Suef province, Egypt. Two tick populations had the highest LC_50_, which was 0.0052% and 0.0051% in Emin elaros and Aldiabia, respectively. Due to the lack of a susceptible cattle tick strain (*R. annulatus*) for comparison, we used published results from previous studies in other countries as reference strains.

The present *R. annulatus* isolates, which were suspected to be ivermectin resistance, had higher LC_50_, and their concentration-mortality slopes were similar to the resistant *R. microplus* tick populations from different areas around the world, including Yucatán [12], Veracruz [15], Sao Paulo [6], and Colombia [6]. A previous study in Mexico [12] indicated that field populations of *R. microplus* were found to be resistant to ivermectin (i.e., LC_50_ of these population was higher than that of the susceptible strains, 0.0048). Consequently, the calculated LC_50_ was 0.00052% and 0.00029% in Alhalabia and Beshnna, respectively, indicating that LC_50_ was lower than that in the other areas. Moreover, the LC_50_ in Alkom was higher (0.0011%) than in Alhalabia and Beshanna, indicating that tick populations from Emen elaros and Aldiabia could tolerate the highest dose of ivermectin. Besides, *R. annulatus* populations from Emen elaros and Aldiabia, which were resistant to ivermectin, had LC_90_ (0.0092% and 0.0084%) higher than *R. annulatus* populations from Alhalabia and Beshnna, which were more susceptible and died at lower concentrations of ivermectin (0.001%). Meanwhile, the LC_50_ for susceptible isolates of *R. microplus* was reported in different parts of the world, including 3.41 ppm (KUL) in India [21], 5.6 ppm in Germany [22], and 4.2 ppm in Brazil [12]. This is higher than that reported in the present study.

Therefore, the tick populations from Emen elaros and Aldiabia areas were resistant to ivermectin. Additionally, Emin elaros and Aldiabia tick populations exhibited the lowest slope. This result coincides with the results of Klafke [2], who argued that data heterogeneity and low slope were associated with high LC_50_ and LC_90_ values, suggesting that many individuals have resistant type allele. A previous study reported that a susceptible strain consisted of completely susceptible individuals and produced the highest slope for the regression line of the dose-response data [12]. Furthermore, it has been stated that low-slopes for the concentration mortality lines, referred as a result of a large heterogeneity with different levels of resistance in the field populations, are studied, and no studies on ivermectin resistant strain have been described with high slopes [6]. The development of *R. annulatus* resistance to chemicals is influenced by environmental, biological, and management factors that might play crucial roles to generate resistance in the tick populations in Beni-Suef province. However, macrolactones have a more rapid selection of resistant ticks by extending their exposure to sub-therapeutic levels of macrolactones. Intriguingly, *R. annulatus* has a short life cycle (four tick generations annually) [23]. The latter suggested that *R. annulatus* might be exposed to acaricides for a long time. The growth of ivermectin resistance has resulted in the widespread use of ivermectin for ectoparasites (ticks) and endoparasites (nematodes), with a broad range of resistance possibility.

The larval immersion test data were matched with the history of ivermectin use at the five study areas. Ivermectin, which is used to control tick and endoparasite infestations, was commonly used in our study sites in Aldiabia and Emenalaros. However, in the other three study areas, ivermectin use is not common. Therefore, the tick populations from Aldiabia and Emenalaros developed resistance to ivermectin. This finding is consistent with findings from San Jeronimo and Tarso farms, Brazil [24], and Colombia [6].

## 5. Conclusions

In conclusion, *R. annulatus* resistance to ivermectin is common with different levels in cattle farms in Beni-Suef province, Egypt. However, the intensive use of macrocyclic lactones to control endo- and ectoparasites in the studied area posed more serious complications of ivermectin resistance, failing in terms of treatment and control.

## Figures and Tables

**Table 1:** Lethal concentration 50 (LC_50_) and LC_90_ of 14-day-old larvae treated with ivermectin using a larval immersion test.

Location/100 Larvae	Slope (SD) *	X2 **	t for Slope ***	LC_50_ (95% CI)	LC_90_ (95% CI)
Alhalabia	1918.292485	78.469074	2.609584	0.000524 (0.000175 to 0.000881)	0.001097 (0.00056 to 0.001888)
Beshnna	1113.815719	13.704343	4.840257	0.000286 (0.000062 to 0.000498)	0.001272 (0.000779 to 0.00188)
Alkom	705.544156	24.025441	4.823858	0.001091 (0.000633 to 0.001573)	0.002648 (0.001585 to 0.003846)
Emin elaros	278.371314	23.13296	6.079659	0.005189 (0.003788 to 0.006676)	0.009269 (0.006482 to 0.012057)
Aldiabia	330.039898	9.684015	12.838334	0.005093 (0.004663 to 0.005601)	0.008422 (0.007669 to 0.009411)

* SD = standard deviation, ** = Chi-square, *** Regression line.

**Table 2:** The mortality rate of larvae treated with different concentrations of ivermectin in the surveyed areas.

		Sample Site					
Concentration		Alkom	Alhalabia	Beshnna	Aldiabia	Eminelaros	* *p*
**Ivermectin concentration %**	0.01	100.0 ± 0.0	100.0 ± 0.0	100.0 ± 0.0	94.00 ± 2.082	87.67 ± 1.453	0.0087 **
	0.005	96.67 ± 0.8819	99.67 ± 0.3333	100.0 ± 0.0	50.33 ± 1.453	54.33 ± 2.333	0.0112 *
	0.0025	90.33 ± 1.453	98.33 ± 0.8819	96.67 ± 0.8819	21.00 ± 2.082	27.67 ± 1.453	0.0115 *
	0.001	54.33 ± 2.333	90.00 ± 0.0	91.00 ± 2.082	6.667 ± 0.8819	8.000 ± 1.155	0.0138 *
	0.0005	33.33 ± 2.028	50.00 ± 0.0	57.33 ± 1.453	5.000 ± 1.155	6.667 ± 0.8819	0.0105 *
	0.000125	18.67 ± 0.6667	19.00 ± 1.000	44.33 ± 2.333	0.3333 ± 0.3333	1.333 ± 0.8819	0.0139 *
	0.0000625	10.67 ± 0.6667	9.333 ± 0.6667	34.00 ± 2.082	0.0 ± 0.0	0.0 ± 0.0	0.0093 **
Negative control		5 ± 2	6 ± 3	5 ± 1	7 ± 3	5 ± 2	-

All tests were conducted in triplicate; * standard deviation; **, superscript letter is extremely significant.
